# Proceedings of the Essex North District Medical Society

**Published:** 1879-01

**Authors:** 


### PROCEEDINGS OF THE ESSEX NORTH DISTRICT MEDICAL SOCIETY.

The quarterly meeting of this society was held in Haverhill, October 23rd., Dr. W. H. Kimball, of Andover, President, in the chair.

Dr. G. M. Garland, of Boston, gave a demonstration of the system of pneumono-dynamics, so fully set forth in his book.

An interesting paper on the Embryology of the Lungs was read by Dr. C. D. Hunkin, of Haverhill; it was illustrated by microscopical specimens of Dr. Hunkin's own preparation. The following is an abstract:

The embryological study of the lungs is best made from microscopic sections of the embryos procured from the eggs of the hen, as these are by far the easiest to obtain. The impregnated hen's eggs are to be placed in an artificial breeder. At the fiftieth or sixtieth hour of their development, they are to be opened, and the embryos removed. The embryos are to be hardened in absolute alcohol, and the sections washed in distilled water. After staining in a dilute solution of carmine they are to be made translucent by means of glycerine.

Of the three layers of cells by which the lungs of the embryo are surrounded, that called the middle is the most important for our consideration, since with the exception of the epithelium of the pleuræ and the cylindrical epithelium of the bronchi, it builds the substratum for the collective tissues of the lungs and pleuræ. A section on the level of the heart of an embryo, prepared as above, under the microscope, discloses the first traces of the lungs as a pair of protuberances, lying symmetrically on both sides of and projecting from the primitive intestine, the so-called *Kopfdarm* of Remark. From an embryological stand-point, the lungs may be regarded as a thickened layer of the primitive vertebral cells,—as a double organ arranged uniformly on both sides of the intestine. . . The epithelium of the lungs, since it plays not an inconsiderable role in the pathological conditions to which the lungs are liable, is worthy of consideration. In choosing the material for microscopic examination of the lung epithelium, the lungs of a dog, inasmuch as in them the alveoli are stronger and the epithelium is larger than in those of many other mammals, will be found most suitable.

The following are some of the points settled by investigation of recent date:

First. The normal lung alveolus has, during the extra-uterine life, a layer of epithelium which is continuous with the rest of the cells covering the bronchi and their branches.

Second. All epithelial forms are represented in the lungs.

Third. The cubic cells of the embryo alveolus may, without undergoing fatty degeneration, with the early acts of respiration, change to a form of polyhedral, flattened epithelium. The lungs have not a form of epithelium peculiar to themselves,—the amount of space in the alveoli at all times determining the form and size of the epithelum.

Fourth. The tissues of the lungs are liable to the same pathological conditions as similar tissues are in other parts of the body.

